# Decreasing the Adverse Effects in Pelvic Radiation Therapy: A Randomized Controlled Trial Evaluating the Use of Probiotics

**DOI:** 10.1016/j.adro.2022.101089

**Published:** 2022-10-03

**Authors:** Irini Lazou Ahrén, Maria Bjurberg, Gunnar Steineck, Karin Bergmark, Bengt Jeppsson

**Affiliations:** a Probi AB, Ideon Science Park, Lund, Sweden; b Department of Hematology, Oncology, and Radiation Physics, Skåne University Hospital, Lund University, Lund, Sweden; c Division of Clinical Cancer Epidemiology, Department of Oncology, Institute of Clinical Sciences, Sahlgrenska Academy at the University of Gothenburg, Gothenburg, Sweden; d Division of Clinical Cancer Epidemiology, Department of Oncology and Pathology, Karolinska Institutet, Stockholm, Sweden; e Department of Surgery, Lund University, Lund, Sweden

## Abstract

**Purpose:**

The aim of this randomized controlled trial was to evaluate the potential benefit from 2 probiotic bacteria of the species *Lactiplantibacillus plantarum* against radiation therapy–induced comorbidities.

**Methods and Materials:**

Women (>18 years of age) scheduled for radiation therapy because of gynecologic cancer were randomly allocated to consume placebo or either low-dose probiotics (1 × 10^10^ colony-forming unit/capsule twice daily) or high-dose probiotics (5 × 10^10^ colony-forming unit/capsule twice daily). The intervention started approximately 1 week before the onset of radiation therapy and continued until 2 weeks after completion. During this period the participants were daily filling in a study diary documenting the incidence and severity of symptoms, intake of concomitant medication, and stool consistency. The primary endpoint was the probiotic effect on the mean number of loose stools during radiation therapy.

**Results:**

Of the 97 randomized women, 75 provided data for the analysis of the results. The mean number of loose stools (sum of Bristol stool type 6 and 7) was not significantly reduced in the probiotic groups, but there was a significant reduction in the mean number of days with >1 loose stool with 15.04 ± 8.92 days in the placebo and 8.65 ± 5.93 days in the high-dose probiotics group (*P* = .014). The benefit was even more pronounced in the 2 weeks following the end of radiation therapy (*P* = .005). Moreover, intake of the probiotics resulted in a reduced severity of the symptoms grinding abdominal pain (*P* = .041) and defecation urgency (*P* = .08) and a reduced percentage of days with these symptoms (*P* = .023 and *P* = .042, respectively), compared with placebo. There were no differences regarding reported adverse events.

**Conclusions:**

Intake of the 2 probiotic bacteria was beneficial and reduced many measures or symptoms of the radiation-induced toxicity in women treated for gynecologic cancer.

## Introduction

Approximately 50% of all patients with cancer receive radiation therapy^1^ that is important in both the curative and palliative treatment of the disease. However, the toxic effect of this treatment on the surrounding normal healthy tissue remains a problem.

The high proliferation rate of the gut epithelium makes it vulnerable to changes and injury after exposure to radiation^2^ and, therefore, pelvic radiation therapy is almost invariably accompanied by acute intestinal inflammation. This is often followed by a progressive fibrosis, months to years later, with an increased risk for stricture formation and intestinal obstruction, a serious problem requiring complex surgical strategies.^3^^,^^4^

Abdominal pain, diarrhea, intestinal obstruction, urgency, fecal incontinence, and malabsorption are some of the symptoms described by the patients^5^ and are part of the so-called pelvic radiation disease.^6^ The severity of the acute bowel toxicity may predetermine the degree of chronic bowel changes and reducing the acute toxicity would, therefore, have a positive effect on the symptoms and quality of life of the patients after the end of treatment. Moreover, considering the positive fact that the improved quality in cancer-treatment regimens results in a higher long-term survivability of cancer patients, it becomes even more important to alleviate the acute radiation-induced toxicity with the aim to support a better posttreatment quality of life for these patients.

It has been shown that the barrier function of the intestinal epithelium is markedly disturbed in the colon following external radiation therapy in patients,^7^ and there are reports about significant changes in the gut microbiome after irradiation, with individuals that suffer the most from treatment-induced diarrhea presenting with the highest reductions in gut bacterial diversity.^8^, ^9^, ^10^, ^11^

Altering the luminal milieu by increasing intake of lactobacilli in experimental animals decreases bacterial translocation and reduces inflammation.^12^ It is reasonable to assume that restoration of the barrier function in radiation therapy may alleviate both acute and late side effects. Germ-free mice are markedly resistant to lethal radiation enteritis,^13^ indicating that gut microbes affect the radiosensitivity of epithelial cells and microbial organisms may suppress or add factors that mediate tissue radiosensitivity.^14^ Therefore, changes in the composition of the intestinal microbiota may be clinically useful to increase the resistance of the gut to radiation therapy. Antibiotics have been used in human clinical trials to manipulate the radiosensitivity of the intestine, but no clearly effective protocol has emerged.^15^

The idea of conditioning the intestinal tissue with probiotic bacteria aiming to mitigate the irradiation-induced injury of the gut epithelium and the symptoms in the pelvic area is a promising approach.^16^, ^17^, ^18^, ^19^, ^20^, ^21^ Possible probiotic mechanisms of action that could be of benefit against radiation- and chemotherapy-induced gastrointestinal discomfort include increased production of the protective mucus on the intestinal epithelial cells, enhanced intestinal barrier function resulting in reduced gut permeability and inflammation, and modulation of the gut microbiota aiming to maintain intestinal homeostasis.

It may be possible to precondition the intestinal mucosa with probiotics beforehand to better withstand noxious insults of the radiation. The aim of the current randomized controlled trial was to evaluate the potential benefit against radiation therapy–induced symptoms and comorbidities from 2 well-characterized probiotic bacterial strains of the species *Lactiplantibacillus plantarum.*

## Methods and Materials

### Design of the study

The study was randomized, double-blind, and placebo-controlled with the objective to evaluate the benefit of *Lactiplantibacillus plantarum* HEAL9 (LPHEAL9; DSM 15312) combined with *Lactiplantibacillus plantarum* 299 (Probi Plantarum 6595; DSM 6595) in individuals scheduled for radiation therapy in the pelvis. The study was conducted at 2 sites in Sweden, both tertiary referral centers (ClinicalTrials.gov identifier: NCT02351089), and ethical approval was received by the ethics committee in Lund, Sweden. All participants provided signed informed consent before randomization into 1 of the 3 study groups. The recruitment was initiated in March 2015 and was completed in December 2018. This clinical study was performed in compliance with the Declaration of Helsinki as well as the International Conference on Harmonisation–Good Clinical Practice guidelines and European Union recommendations (CPMP/ICH/135/95).

### Study participants

The study population was women older than 18 years with a diagnosis of gynecologic cancer and scheduled for external beam radiation therapy at a minimum dose of 40 Gy to the pelvis. The radiation therapy was either a primary or secondary adjuvant treatment following surgery, and concomitant chemotherapy could be part of the standard-of-care treatment. The exclusion criteria were previous irradiation in the pelvic area or reluctancy to refrain from using other probiotic products during participation in the study. The radiation therapy was administered in accordance with the national guidelines for the particular diagnoses, with volumetric modulated arc therapy technique and with 1.8 to 2.0 Gy per fraction. The bowel bag was contoured according to the Radiation Therapy Oncology Group criteria with a soft constraint of V30 Gy for ≤300 cc. Brachytherapy was allowed as part of the treatment.

### Study procedures

The study participants were recruited at the respective oncology clinics. Intake of the investigational product was initiated 1 to 2 weeks before the start of the scheduled radiation therapy and continued throughout the treatment until 2 weeks after the end of radiation therapy. The length of the radiation therapy period varied among participants (23-36 days). During their participation in the study, all participants were asked to daily fill in a diary with questions related to their gastrointestinal health, and they were also asked twice during the study to fill out a more detailed questionnaire related to their quality of life. At randomization, study-related visits to the clinics were scheduled for the start of radiation therapy (1-2 weeks after randomization) and at the end of the study (2 weeks after the last radiation therapy session). A telephone contact was scheduled halfway through the study to remind the participants about the daily intake of the investigational product and the study diary.

### Investigational product

The active investigational product consisted of *Lactiplantibacillus* (former *Lactobacillus) plantarum* HEAL9, hereafter called *L. plantarum* HEAL9, and *Lactiplantibacillus* (former *Lactobacillus) plantarum* 299, hereafter called *L. plantarum* 299. Each bacterial strain was equally represented in the total dose of either 1 × 10^10^ colony-forming unit (CFU)/capsule (low-dose probiotic group; LDP) or 5 × 10^10^ CFU/capsule (high-dose probiotic group; HDP). The placebo was of identical appearance, taste, and texture as the active product, excluding the bacteria. The investigational product was supplied in capsules containing a powder with freeze-dried bacteria and maltodextrin as filler. The capsules should be ingested twice daily: 1 capsule in the morning and 1 in the evening. The participants were stratified into 2 groups: those who received only radiation therapy and those with concomitant chemotherapy. Within each of these groups, participants were randomly allocated to receive HDP or LDP product or placebo based on a computer-generated randomization list with blocks of 3 and the ratio of 1:1:1, respectively (ie, they were given the next available randomization number from the corresponding list). Sealed envelopes were prepared for the allocation concealment and were safely stored by the investigators throughout the study. The labeling of the study product and the preparation of the sealed code envelopes were done by personnel not otherwise involved in any study-related activities. Both study participants and investigators were blinded to the identity of the study product.

### Outcomes

The primary objective of the study was to investigate the possible benefit from using *L. plantarum* HEAL9 and *L. plantarum* 299 compared with placebo on the mean number of loose/watery stools reported by the study participants (also presented as severity of loose stools). Loose/watery stools were defined as the sum of types 6 and 7 based on the Bristol stool scale that was included in the study diary (a copy of the Bristol stool chart is found in Figure E1). The secondary objectives included the evaluation of the probiotic effect on the incidence (ie, participants reporting the symptom), frequency (ie, days with the symptom), and severity of abdominal gas, feeling sick, defecation urgency, fecal leakage, abdominal cramp/convulsion, grinding abdominal pain, presence of mucus in feces, rectal discharge of mucus, and the need to use rescue medication for diarrhea, constipation, or abdominal pain. These symptoms were chosen based on the clinical experience of the investigators and previously published data about the conditions that patients irradiated in the pelvic area may suffer from.^22^ The severity of the secondary endpoints was defined as number of occasions with the symptom except for the experience of abdominal gas and feeling sick that were rated on a 4-point subjective scale (none = 0, little = 1, moderate = 2, severe = 3). If not otherwise stated, the analysis of the primary and secondary objectives was done for the time-period from day 8 after the first radiation therapy session until the last day with irradiation. All endpoints were assessed based on the information provided by the participants and the investigators in the study related documents (diary and Case Report Form)*.*

### Sample size

The study was planned to be conducted in 2 phases. The first one was 3-armed including the placebo and 2 probiotic groups (HDP vs LDP). The second phase would include 1 of the probiotic groups based on the results from an interim analysis and the placebo. The sample size in the first phase was calculated based on the expected/possible number of loose stools per day. To detect a difference in the mean number of daily stools of ≥1 between placebo and active probiotic group, assuming a standard deviation of 1.75 and a significance level of 5% in a 1-sided test, a sample size of 38 participants per group would be needed. The interim analysis was conducted when approximately 20 participants per group had completed the study using data for the primary objective. The study participants, the investigators and the sponsor remained blinded to the randomization throughout this process. The interim analysis indicated that to achieve 80% power to get a 2-sided *P* value <5% when testing the primary objective, 71 evaluable participants per group were needed in the placebo and the HDP group. However, due to difficulties with the recruitment pace, the study was ended shortly after the communication of the interim analysis results. At this stage there were 75 evaluable participants randomized in the study.

### Statistical analysis

The statistical analysis was performed using the StatXact version 11.1.0 and STATA version 16. The nonparametric Mann-Whitney *U* test was applied for the analysis of continuous variables, whereas the Fisher exact test was used for the categorical endpoints. For the statistical analysis all participants within the same intervention group were pooled and analyzed together. The efficacy analysis set included all participants that provided diary data for the primary endpoint during at least 14 days after the first radiation therapy session. All participants having consumed at least 1 dose of the investigational products were included in the analysis of safety parameters. All presented *P* values are nominal, that is, not adjusted for multiplicity and *P* values <5% are considered statistically significant.

## Results

**Figure 1:**
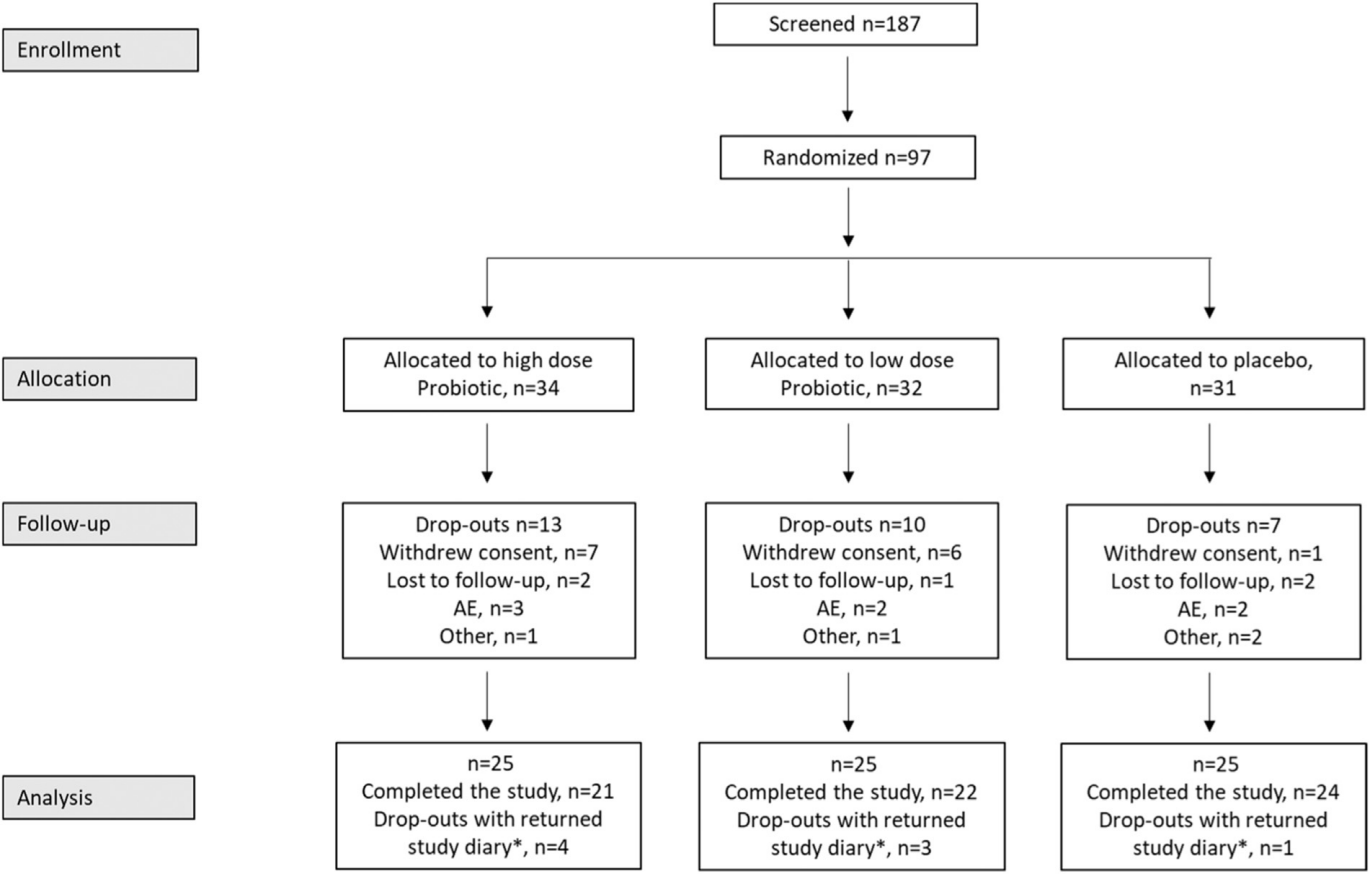
Flowchart for the clinical study. *Participants that withdrew their consent but returned a study diary with data for at least 14 days after the start of radiation therapy were included in the statistical analysis. *Abbreviation:* AE = adverse events

Following the screening of 187 women diagnosed with gynecologic cancer and scheduled for radiation therapy, there were 97 participants in total (Fig. 1) that were randomly allocated into 1 of the study groups to consume either HDP (n = 34), LDP (n = 32), or placebo (n = 31). The screening failures were primarily explained by the reluctancy to refrain from using other probiotic products during the study. There was a relatively high drop-out rate (31%), not statistically significant among the groups, with 13 and 10 women in the HDP (38.2%) and LDP (31.3%) groups, respectively, and 7 women in the placebo group (22.6%). Most of the dropouts were due to withdrawn consent. However, there were 8 participants among the dropouts that returned a partially completed study diary, that is, data for at least 14 days from the start of radiation therapy that could be included in the statistical analyses.

As described in Table 1, the groups were similar in terms of age, body weight, smoking habits, medical history, external radiation dose received, and the number of participants that had chemotherapy concomitant to the radiation therapy. In general, half of the women had cervical cancer and the other half had corpus cancer.. The number of planned irradiation sessions varied from 23 to 36, and there was no difference among the groups.

**Table 1:** Baseline and demographic data presented as mean ± standard deviation for categorical variables and as absolute values (%) for nominal variables

Characteristic	Placebo n = 25	Probiotic low n = 25	Probiotic high n = 25
**Age (y)**	56.4 ± 16.1	60.1 ± 14.1	55.0 ± 16.5
**Body weight (kg)**	76.3 ± 17.9	78.1 ± 20.3	70.0 ± 11.3
**Body mass index (kg/m^2^)**	27.7 ± 5.4	28.3 ± 6.8	25.8 ± 4.1
**Smoking**			
Current smoker	0 (0%)	3 (12%)	2 (8%)
Former smoker	9 (36%)	9 (36%)	13 (52%)
**Previously received related treatments**			
Any treatment	13 (52%)	14 (56%)	18 (72%)
Surgery	16 (64%)	17 (68%)	19 (76%)
Chemtherapy	9 (36%)	9 (36%)	14 (56%)
**Type of cancer**			
Cervix	10 (40%)	12 (48%)	14 (56%)
Corpus	14 (56%)	12 (48%)	10 (40%)
Vaginal	1 (4%)	1 (4%)	0 (0%)
Vulva	0 (0%)	0 (0%)	1 (4%)
**Total dose of external irradiation (Gy)**	53.41 ± 8.93	52.20 ± 9.18	49.18 ± 11.28
**Subgroups based on dose of external irradiation (Gy)**			
40-49	12 (48%)	10 (40%)	11 (44%)
50-59	4 (16%)	9 (36%)	9 (36%)
≥60	9 (36%)	6 (24%)	5 (20%)
**Received brachytherapy in addition to external radiation**			
Yes	10 (40%)	12 (48%)	12 (48%)
**Received concomitant chemotherapy**			
Yes	13 (52%)	15 (60%)	12 (48%)

There are no statistical differences between the probiotic groups and the placebo.

### Loose stools during and after radiation therapy

**Figure 2:**
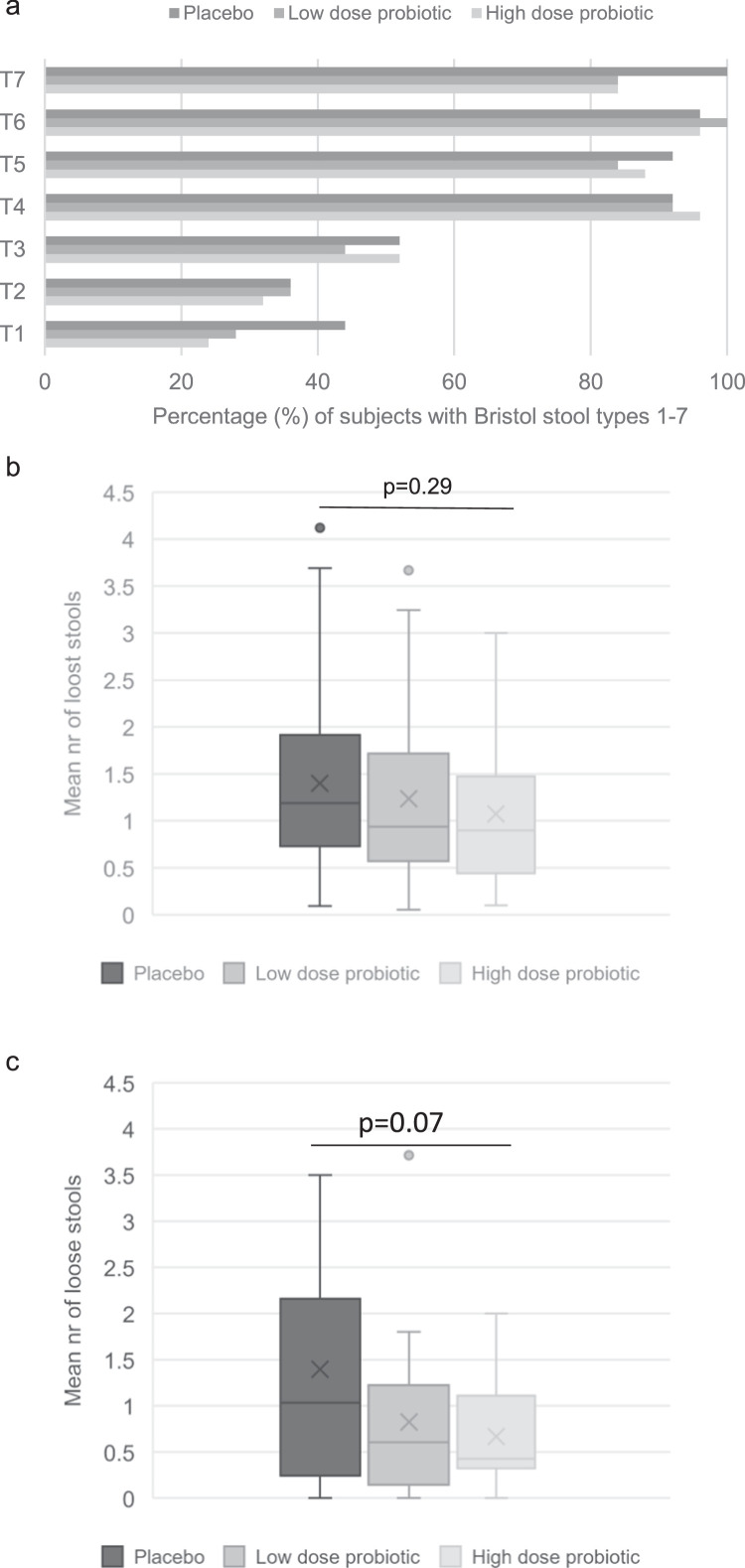
Incidence of Bristol stool types 1 to 7 (A) and mean daily number of loose stools for the period from day 8 until the last day with radiation therapy (B) or during the 14 days after radiation therapy (C).

**Figure 3:**
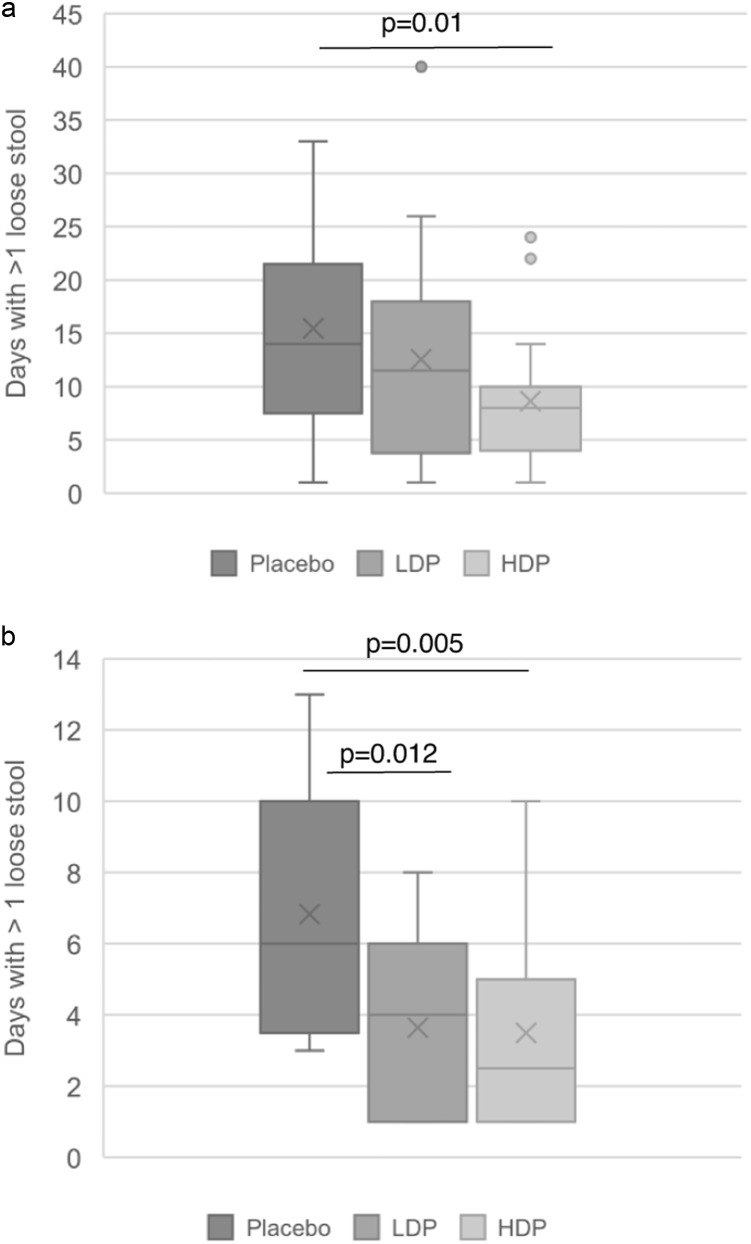
Absolute number of days with >1 loose stool from start to end of radiation therapy (A) and during the 14 days after radiation therapy (B). *Abbreviations:* HDP = high-dose probiotic; LDP = low-dose probiotic.

Almost all the participants experienced loose stools during their radiation therapy with the lowest incidence being 84% of the participants in the probiotic groups reporting Bristol stool type 7 compared with 100% of the participants in the placebo group (*P* = .1; Fig. 2A). The mean daily number of loose stools, from day 8 post start of radiation therapy until the last day with radiation therapy, was lower in the HDP group (1.06 ± 0.75, *P* = .3) compared with placebo (1.40 ± 1.05) but with no statistical significance (Fig. 2B**)**. However, in line with the results from the interim analysis, 71 participants per group were needed to show a significant difference between the probiotic groups and the placebo. The probiotic benefit on the severity of loose stools was more prominent during the 14 days after the end of radiation therapy (Fig. 2C). In an analysis focusing on days with >1 loose stool per day, there was a significant difference between the HDP group and the placebo group in terms of number of days with loose stools during the whole period with radiation therapy (Fig. 3A). There were 20 of 25 participants in the HDP group that reported a mean of 8.65 ± 5.93 days with >1 loose stool and 21 of 25 participants in the placebo group with 15.04 ± 8.92 days (*P* = .014). Individuals that terminated the study earlier were not included in this analysis. The results were similar also during the 14 days after radiation therapy (Fig. 3B). There were 16 participants (76.2%) in the HDP group with a mean of 3.78 ± 2.76 days with >1 loose stools and 17 participants (70.8%) in the placebo group with 6.82 ± 3.45 days (*P* = .005). In the LDP group, there were 17 participants (77.3%) with a mean of 3.64 ± 2.48 days with >1 loose stool (*P* = .012 compared with placebo).

### Stool types during radiation therapy

There was a significantly higher daily mean number of stool type 4 in the HDP group compared with placebo with a score of 0.59 ± 0.58 versus 0.32 ± 0.32, *P* = .03, respectively, and a corresponding lower nr of stool type 6 (0.48 ± 0.35 vs 0.79 ± 0.72; *P* = .06, Fig. E2). The groups were similar in terms of incidence of each stool type except for the lower incidence of type 7 in the 2 probiotics groups compared with placebo (Fig. 2A). The percentage of days with each stool type was also similar in the study groups except for type 5, which was reported significantly more days in the HDP group compared with placebo (37 ± 24% vs 21 ± 16% of days with radiation therapy; *P* = .01).

### Gastrointestinal symptoms during radiation therapy

There was a significant benefit from the usage of the probiotic product on the severity of grinding abdominal pain (Table E1). The mean daily severity score reported in the placebo group was 0.88 ± 1.64 compared with 0.38 ± 0.60 (*P* = .057) in the HDP and 0.41 ± 0.99 (*P* = .041) in the LDP group. A pooled analysis of the 2 probiotic groups together supported further the observed probiotic benefit for this symptom (*P* = .02 compared with placebo). The severity score for the symptom defecation urgency was reduced compared with placebo, especially with the LDP product (0.95 ± 0.73 vs 0.63 ± 0.76 respectively; *P* = .08). The symptom abdominal cramp was also slightly improved after intake of the probiotic product (*P* = .12). No differences were identified between the probiotic groups and the placebo with regards to the severity of the other gastrointestinal symptoms.

**Figure 4:**
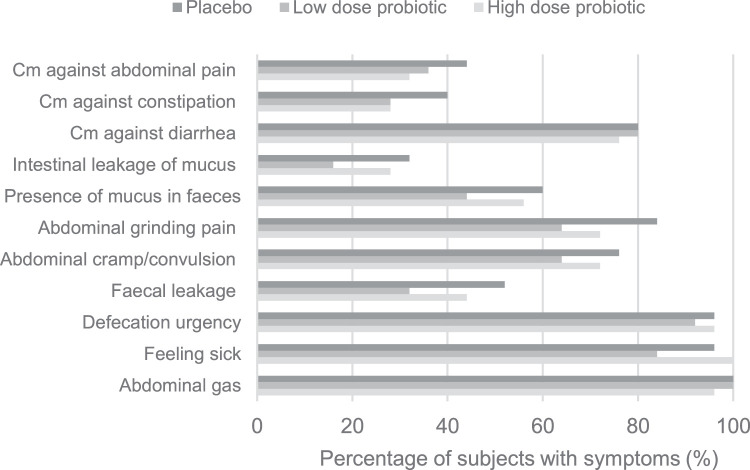
Percentage of participants in each group reporting at least 1 day with each of the secondary symptoms.

Intake of the probiotics reduced the frequency of the symptoms feeling sick, defecation urgency, abdominal cramp/convulsion, and grinding abdominal pain. The frequency is presented as the percentage of days that a symptom was reported from day 8 until the last day with radiation therapy (Table E2). The symptom defecation urgency was reported in the placebo for 61 ± 32% of the days compared with 29 ± 18% in the LDP group (*P* = .042). Likewise, the percentage of days with grinding abdominal pain was reduced from 35 ± 33% in the placebo to 18 ± 0.29% in the LDP group (*P* = .023) and 20 ± 25% in the HDP group (*P* = .087). Furthermore, the participants in the LDP group experienced fewer days with feeling sick (*P* = .06) and abdominal cramp/convulsion (*P* = .065) compared with placebo. No differences were identified in the study groups in terms of incidence of the gastrointestinal endpoints (Fig. 4).

### Usage of concomitant medication

There was no difference between the probiotic groups and the placebo group with regards to usage of concomitant medication (Tables E1 and E2, Fig. 4). The most common concomitant medication was against diarrhea with an incidence of approximately 80% in all the study groups. Medication against constipation or abdominal pain was used by 20% to 40% of the participants. A small difference was seen with regards to the time from start of radiation therapy until the first usage of rescue medication, especially for antidiarrheals. Presented as percentage of days in the study from start until the first intake of medication there was a mean of 51 ± 37% of days in the HDP group compared with a mean of 38 ± 38% in the placebo group (*P* = .17). The corresponding absolute values showed that for the 19 participants (76%) in the HDP group that used concomitant medication against diarrhea, they initiated the treatment on day 17 ± 8.4 compared with day 14.3 ± 8.6 for the 20 participants (80%) in the placebo group*.*

### Change in body weight from baseline to end of study

A reduction in body weight from baseline to end of study was reported by the participants in all the study groups. The least reduction was seen in the HDP group with a mean change within the group of –0.57 ± 2.53 kg (*P* = .51), whereas there was a significant reduction by –2.54 ± 3.33 kg in the LDP group (*P* = .037) and –1.33 ± 4.10 kg in the placebo (*P* = .061). However, the interpretation of these results should be done cautiously because there were missing values in all 3 study groups for the actual body weight at end of study (data was collected for approximately 40% of the participants in the probiotic groups and 64% in the placebo group).

### Safety data

The groups were similar in terms of reported adverse and serious adverse events with 32% to 44% of the participants reporting at least 1 adverse event (Table E3). Most of the adverse events were unlikely related to the study product, and in the few cases with possible or probable causality, the problems were associated with abdominal discomfort.

## Discussion

In the present study, the aim was to investigate whether alteration of the microbial milieu by administration of probiotics might improve the resistance of the gut mucosa and reduce side effects to radiation in the pelvic area. We show that intake of the probiotic bacteria *L. plantarum* 299 and *L. plantarum* HEAL9 attenuated the morbidity and supported a faster recovery from gastrointestinal symptoms developed during radiation therapy. The most obvious benefit in the study population of women treated for gynecologic cancer was the reduced number of days with >1 loose stool as well as an improvement of the symptoms grinding abdominal pain and defecation urgency that were milder and were experienced fewer days after intake of the probiotic compared with placebo. Moreover, the data are indicative of a probiotic benefit toward the normalization of the stool consistency and the reduced incidence of loose stools (Bristol stool type 7) but there was not a significant difference between the probiotic groups and the placebo in terms of the primary endpoint. However, this is probably due to the fact that the actual sample size was smaller than originally aimed for. As previously mentioned, difficulties with recruiting the patients resulted in an earlier termination of the trial which may be seen as a limitation of the current study. The heterogeneity of the study population in terms of treatment received, such as the variability in use of chemotherapy and brachytherapy, may also be a limiting factor. More conservative inclusion criteria would though have made the recruitment of study participants even more difficult to pursue.

A systematic review conducted by the Mucositis Study Group of the Multinational Association of Supportive Care in Cancer/International Society of Oral Oncology suggested that probiotic treatment containing *Lactobacillus* spp. may be beneficial for prevention of chemotherapy and radiation therapy–induced diarrhea in patients with pelvic malignancies.^19^ In the previously conducted studies, the probiotic bacteria or placebo have most commonly been administered starting 1 week before the onset of radiation therapy until the end of treatment. In the study by Linn et al,^23^ 54 women treated for cervical cancer consumed either placebo or probiotics 3 times daily. The probiotic group reported a reduced incidence of radiation-induced diarrhea that was 53.8% compared with 82.1% in the placebo (*P* < .05) and a reduced usage of loperamide (*P* < .01). Similar results were also reported by Chitapanaraux et al^24^ in a study with 63 women also treated with radiation therapy for cervical cancer. The women consuming a total of 4 × 10^9^ CFU/d of a combination of a *Lactobacillus acidophilus* and a *Bifidobacterium bifidum* strain had significantly lower incidence of grade 2 to 3 diarrhea (9%) compared with the participants in the placebo group (45%; *P* < .002). Previously published studies with a probiotic application during radiation therapy for cervical cancer^23^, ^24^, ^25^, ^26^, ^27^ studied the probiotic effect on the incidence and severity grade of radiation induced diarrhea (defined by the World Health Organization as 3 or more loose or liquid stools per day or more frequent passage than is normal for the individual) and hence reported about probiotic effects in association with more severe cases of loose stools. In the current study, the focus was instead on changes in the mean number of loose stools as these are defined using the Bristol stool scale and irrespective of the severity grade of the diarrhea. However, days with >1 loose stool were significantly fewer in the probiotic groups compared with placebo. The data indicates that the probiotic benefit may be more prominent the more frequent the radiation-induced comorbidities are. If the treatment-induced comorbidities become relatively less severe in parallel with the improved precision of radiation therapy, larger clinical studies may be needed for a better evaluation of the probiotic benefit in relatively milder cases of radiation disease. Nevertheless, despite the small size of the current study it is a strength that the combination of *L. plantarum* 299 and *L. plantarum* HEAL9 significantly improved many of the endpoints evaluated compared with placebo. There was a normalization of stool consistency, a faster recovery, and improvements in defecation urgency and grinding abdominal pain. To the best of our knowledge, this is the first study on the probiotic benefit against the radiation-induced disease in gynecologic cancers, monitoring this array of symptoms, carefully selected based on epidemiologic data from cancer survivors. In terms of differences in efficacy of the 2 probiotic doses that were evaluated, there is not an obvious dose-dependent probiotic benefit, which is in line with what was previously reported by Demers et al^28^ and Ouwehand et al^29^ when using probiotics for prophylaxis in colorectal cancer and relief of irritable bowel syndrome. It may be that, once a certain level of conditioning of the intestinal mucosa has been achieved, there are no additional benefits from increasing the probiotic dose, at least not for the endpoints that were evaluated with the current study design.

The probiotic intervention was safe, and there were no major adverse events reported. The safe usage of *L. plantarum* 299 and *L. plantarum* HEAL9 has previously been confirmed in multiple clinical studies.^30^, ^31^, ^32^ Both *L. plantarum* 299 and *L. plantarum* HEAL9 survive the passage through the gastrointestinal tract and adhere to the intestinal mucosa through a mannose-dependent mechanism that can result in the competitive exclusion of pathogenic bacteria.^33^, ^34^, ^35^, ^36^ This can support the microbial homeostasis in the irradiation-affected gut and, combined with a strengthened barrier function, may result in fewer loose stools and defecation urgency.

## Conclusions

Summarizing the results from the current study, intake of *L. plantarum* 299 and *L. plantarum* HEAL9 during radiation therapy in the pelvic area supported a better tolerance of the irradiation with benefits reported primarily in relation to stool consistency, defecation urgency, and abdominal pain.

## Appendix Supplementary materials

Image, application 1

## Footnotes

Supplementary material associated with this article can be found in the online version at doi:10.1016/j.adro.2022.101089.
