# Supplementary material for: Decreasing the Adverse Effects in Pelvic Radiation Therapy: A Randomized Controlled Trial Evaluating the Use of Probiotics
Source: Adv Radiat Oncol. 2022 Oct 3;8(1):101089. doi: 10.1016/j.adro.2022.101089 (PMC9723296; doi:10.1016/j.adro.2022.101089)

SUPPLEMENTARY MATERIAL

Figure E1: Bristol Stool Chart

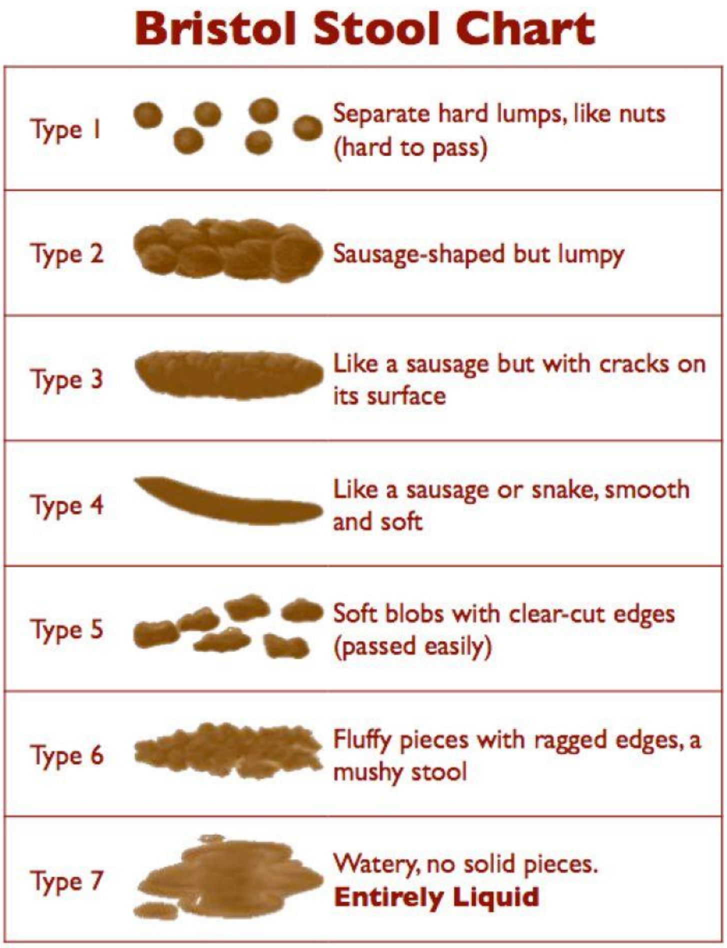

Table E1: Mean severity score per day for the secondary endpoints during the period from day 8 until the last day with radiotherapy.

Symptom group	Placebo n=25	LDP n=25	HDP n=25
Abdominal gas	1.12 ± 0.69	1.09 ± 0.63	1.08 ± 0.74
Feeling sick	0.93 ± 0.66	0.72 ± 0.84	1.13 ± 0.77
Defecation urgency	0.95 ± 0.73	0.63 ± 0.76	0.72 ± 0.53
Faecal leakage	0.31 ± 1.14	0.10 ± 0.26	0.06 ± 0.11
Abdominal cramp/convulsion	0.67 ± 0.95	0.41 ± 1.00	0.37 ± 0.40
Abdominal grinding pain	0.88 ± 1.64	0.41 ± 0.99*	0.38 ± 0.60†
Presence of mucus in faeces	0.49 ± 1.24	0.36 ± 0.88	0.25 ± 0.53
Rectal mucus discharge	0.35 ± 1.22	0.06 ± 0.24	0.05 ± 0.12
Concomitant medication against diarrhea	0.51 ± 0.60	0.46 ± 0.38	0.36 ± 0.45
Concomitant medication against constipation	0.07 ± 0.12	0.13 ± 0.27	0.04 ± 0.08
Concomitant medication against abdominal pain	0.37 ± 0.89	0.20 ± 0.44	0.04 ± 0.09

*p value < 0.05 in comparison to placebo; †p value < 0.1 in comparison to placebo

Table E2: Percentage (%) of days with symptoms measured for the period from day 8 after the start of radiotherapy until the last day with radiation.

Symptom group	Placebo n=25	LDP n=25	HDP n=25
Abdominal gas	74 ± 29	72 ± 31	69 ± 35
Feeling sick	61 ± 32	40 ± 36[†]	68 ± 32
Defecation urgency	45 ± 28	29 ± 18[*]	36 ± 26
Faecal leakage	07 ± 15	0.5 ± 10	0.4 ± 06
Abdominal cramp/convulsion	31 ± 32	15 ± 22[†]	21 ± 24
Abdominal grinding pain	35 ± 33	18 ± 29[*]	20 ± 25[†]
Presence of mucus in faeces	17 ± 26	14 ± 27	13 ± 23
Rectal mucus discharge	0.9 ± 20	0.2 ± 07	0.3 ± 06
Concomitant medication against diarrhea	30 ± 27	26 ± 22	20 ± 23
Concomitant medication against constipation	0.6 ± 10	11 ± 24	0.4 ± 08
Concomitant medication against abdominal pain	16 ± 32	13 ± 31	0.2 ± 04

[*]p value < 0.05 in comparison to placebo; [†]p value < 0.1 in comparison to placebo

Table E3: Adverse and severe adverse events reported in the study. Data present number and percentage of participants in the ITT population, n (%) and number of adverse events.

	Placebo	LDP	HDP
Subjects with AEs	11(44)	8(32)	9(36)
Subjects with 1 AE	7(28)	3(12)	5(20)
Subjects with 2 AEs	3(12)	2(8)	3(12)
Subjects with ≥3 AEs	1(4)	3(12)	1(4)
Total number of AEs	14	17	11
AEs based on causality of the study product			
Unlikely related	9	15	10
Possibly related	2[*]	0	1[†]
Probably related	3[§]	2[**]	0
Subjects with SAEs	4 (16)	3 (12)	2 (8)
Total number of SAEs[††]	5	5	3

[*]feeling sick, constipation; [†]feeling sick; [§]abdominal discomfort, pain in the pelvic area, increased values of liver markers; [**]abdominal pain, oral mucositis; [††]all SAEs but one, were unrelated or unlikely related to the study product. The SAE in the placebo group that was possibly related to the study product was a severe obstipation that was resolved following administration of laxatives. LDP: low dose probiotic; HDP: high dose probiotic

Figure E2: Mean daily number for Bristol stool types 2-7, during the period from day 8 to the last day with radiotherapy

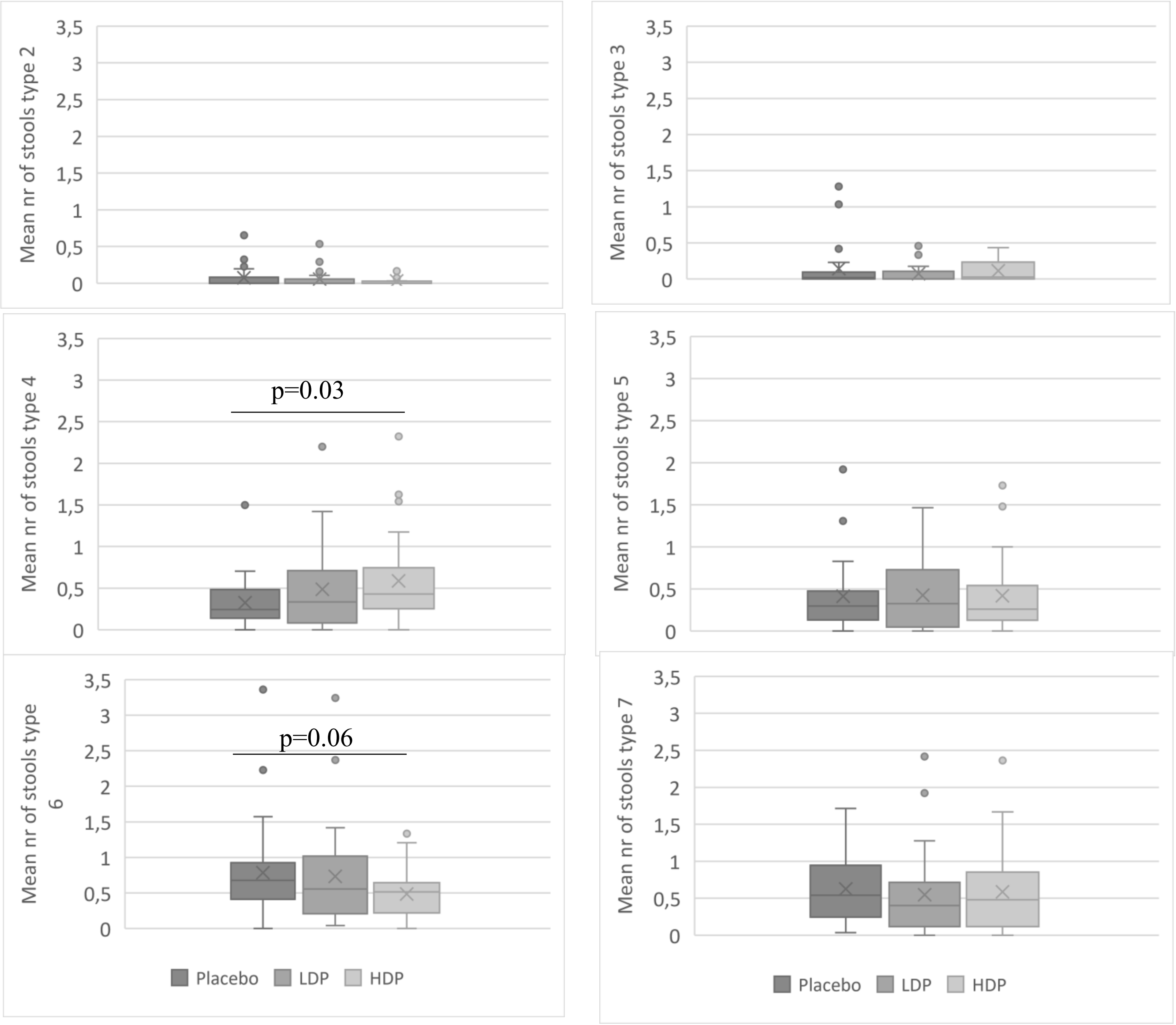

Supplement: Supplementary file 1 [file mmc1.pdf]
